# Mitigating perioperative pressure injuries in microsurgical breast reconstruction

**DOI:** 10.3389/fsurg.2025.1513082

**Published:** 2025-03-05

**Authors:** Amanda Fazzalari, Susanna Gebhardt, Ryoko Hamaguchi, Shailesh Agarwal

**Affiliations:** ^1^ Division of Plastic and Reconstructive Surgery, Brigham and Women’s Hospital, Boston, MA, United States; ^2^ Division of Plastic and Reconstructive Surgery, Lahey Hospital and Medical Center, Burlington, MA, United States

**Keywords:** pressure injury, plastic surgery, breast reconstruction, microsurgery, microvascular breast reconstruction short running head: mitigating PI in micro breast reconstruction

## Abstract

Pressure injuries (PI) that develop in the operating room (OR) account for just under half of all hospital acquired pressure injuries (HAPI) and contribute significantly to the high cost and patient morbidity of HAPI. Microvascular autologous breast reconstruction poses specific risks to PI development in patients and should be addressed by the reconstructive microsurgeon. Standard risk factors for perioperative PI include patient immobility, absent pain perception, and challenges to maintaining normal body temperature while under general anesthesia for surgery. Specific intraoperative risk factors relevant to patients undergoing microvascular autologous breast reconstruction include extended length of surgery and patient repositioning. The risk of PI increases significantly when operative time exceeds 3 h and patient repositioning, with changes in positioning subjecting specific anatomic locations to increased pressure and friction. For these reasons, placement of positioning devices at high-risk anatomical locations is particularly important, such as the use of polyurethane or polyether mattresses, multilayered silicone foam dressings, and gel, foam, or fluidized positioners. The implementation of periodic body positioning checks and clear communication between surgical teams regarding awareness and status of pressure points is helpful in mitigating risk of perioperative PI. Preoperative risk assessments and skin exams may also be useful, as well as postoperative skin exams and early movement out of bed on postoperative day 0 and ambulation on postoperative day 1. These guidelines will reduce the risk of PI development in patients undergoing reconstructive breast surgery.

## Background

**Figure 1:**
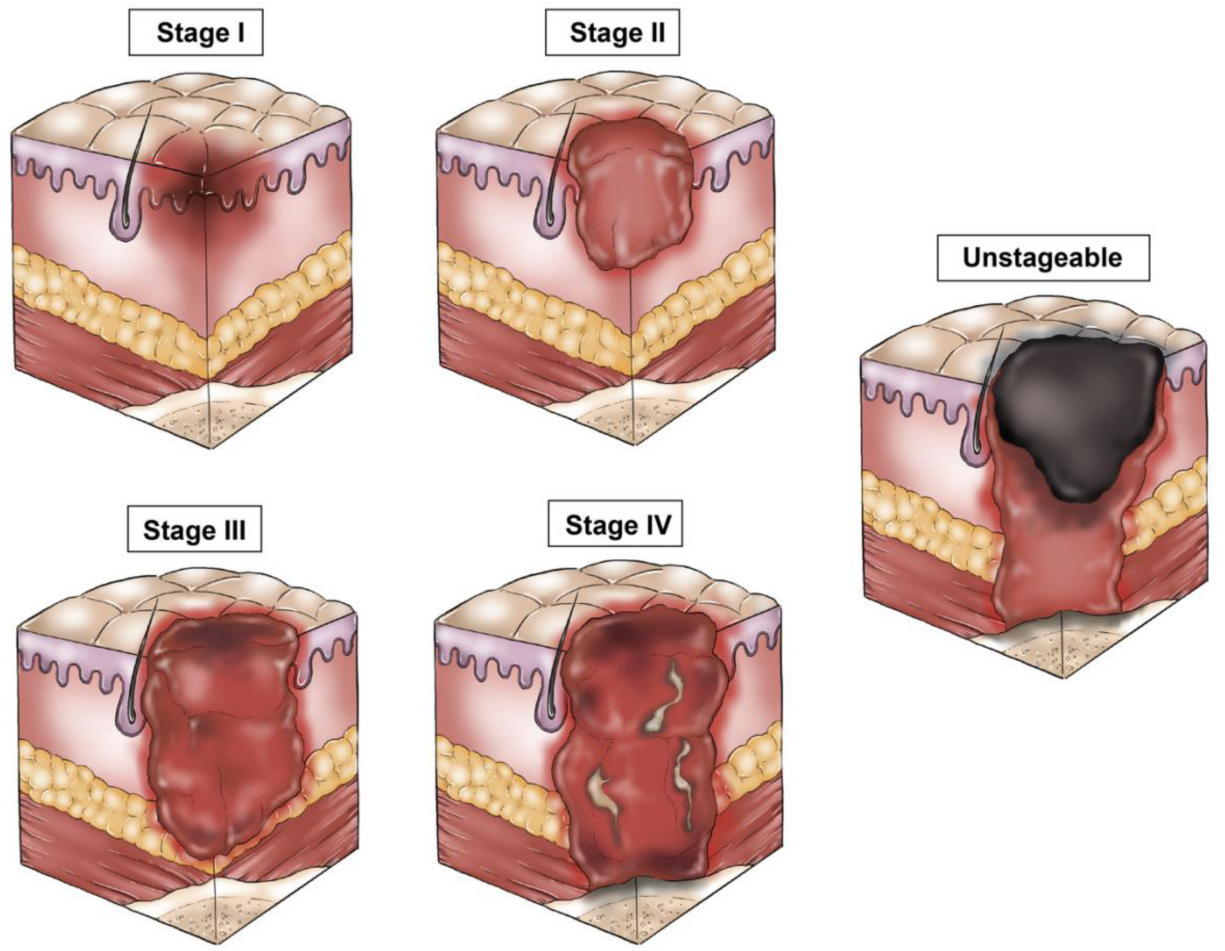
Stages of pressure injury.

Hospital acquired pressure injuries (HAPI) are an extremely common and preventable outcome of surgery and hospitalization, both in the United States and globally. HAPI continue to be responsible for high hospital costs and significant patient morbidity, with greater stages of pressure injury (PI) responsible for greater costs and morbidity (Figure 1). Nationally, the incidence PI ranges from 1 to 3 million annually, with HAPI occurring in 7.5% of all hospitalized patients (1). Intraoperatively acquired PIs are estimated to occur in approximately 12%–66% of patients, with any PI occurring within the 72 h following an operation labeled as a surgery-related PI (2). The costs incurred to the patient include increased length of hospital stay, pain, complications related to delayed wound healing or infection, use of additional inpatient and outpatient resources (nursing care or physical therapy), emotional distress and physical strain on the patients and their care givers (2). Additional monetary costs typically range between $14,000–40,000 per patient, with annual costs estimated at $750 million to $1.5 billion nationally (2). These costs are even more outstanding in the context of the Centers for Medicare and Medicaid Services (CMS) no-pay policy, implemented in 2008, in which “preventable” injuries, including HAPI, do not qualify for reimbursement (3).

PIs developing in the operating room represent up to 45% of all HAPIs and any PI occurring within 72 h after surgery may be the result of an initial insult that occurred intraoperatively (4). As many as 58% of perioperative PIs occur after the 5th hospital day (5), resulting in general underreporting of perioperative PIs (4). In patients undergoing surgery with operative time exceeding 3 h, the rate of PI is estimated to be at least 8.5%, making it a particularly important concern for plastic surgeons performing more complex and thus, lengthy operations (6).

Breast reconstruction remains among the top 5 categories of operations performed by plastic surgeons across the country, accounting for 151,641 procedures in 2022 (7). The Deep Inferior Epigastric Perforator (DIEP) flap remains the gold standard for autologous reconstruction, but as a complex procedure, with many intricate steps involved, it has historically been associated with significantly longer operative times when compared to implant based reconstruction (8). Even under conditions of maximal efficiency the operation may take an average of 4 h (8), but more commonly operative times range from 8 to 9.5 h for unilateral and bilateral reconstructions (9). In patients not candidates for a DIEP flap or other abdominally based microsurgical breast reconstruction (muscle-sparing transverse rectus abdominus myocutaneous flap or superior inferior epigastric artery perforator flap), secondary options include free flaps from the buttock (superior gluteal or inferior gluteal artery perforator flaps) or thighs (transverse upper gracilis or profunda artery perforator flaps) (10). These procedures are similarly complex and lengthy, with average operative times ranging from 7 to 12 h for bilateral procedures (11, 12). Additionally, positioning the patient in the prone, lateral, or frog-legged position, and requiring positioning changes also increase the risk of PI.

**Figure 2:**
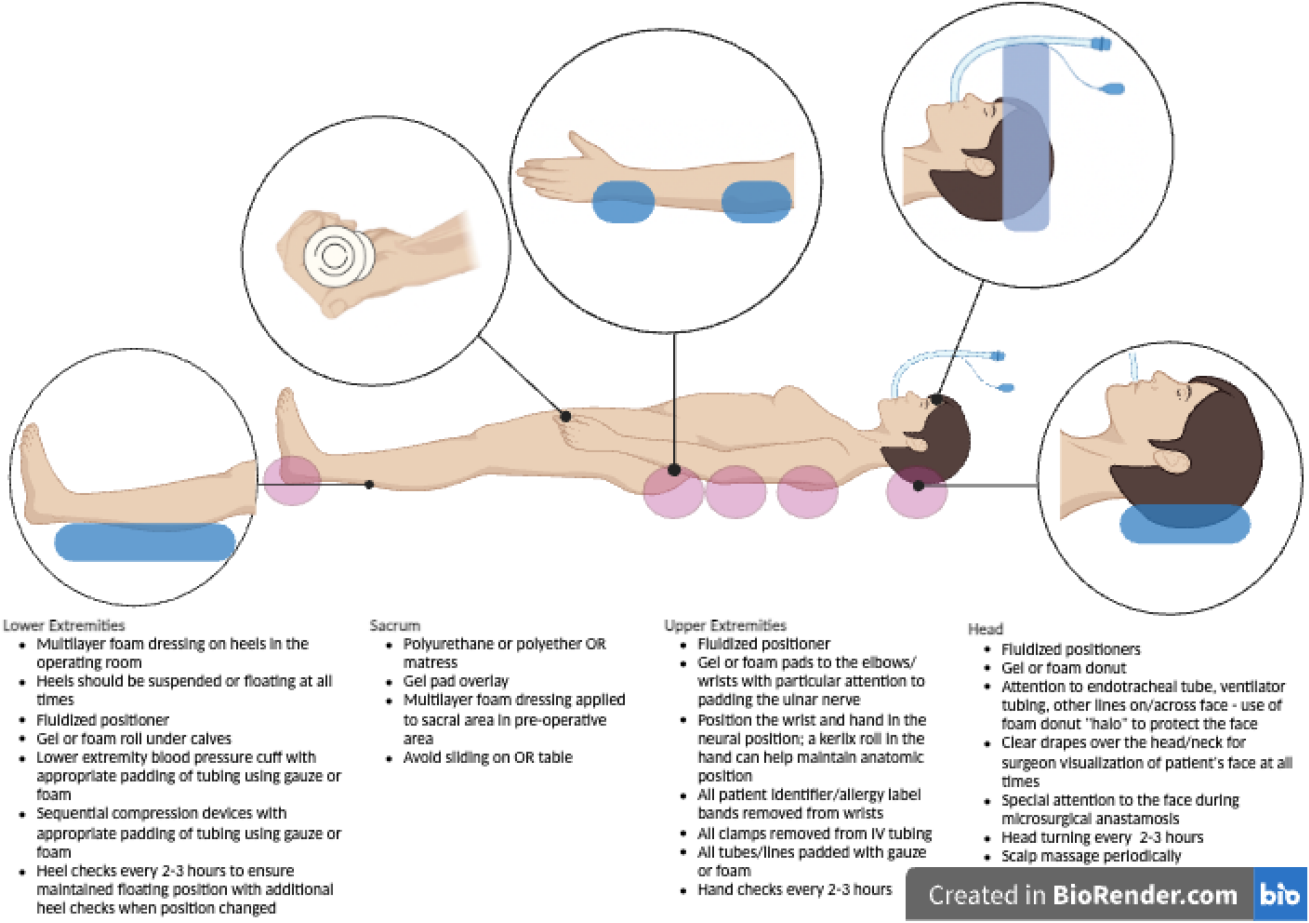
Positioning and padding considerations by anatomic area in the supine position.

PIs acquired in the operating room are clearly an important concern for the reconstructive surgeon and for health systems at large. However, despite the tremendous costs of intraoperative/perioperative PIs to patients and health care system, there is a paucity of publications focusing on perioperative PIs and there is little evidence to suggest best practices for preventing intraoperative PIs. Moreover, there are no papers discussing the risks of PI during microsurgical cases, which are known to have prolonged operative time, position changes, and elevated risk of intraoperative PI. In this review we will discuss the risk factors for PI specific to the patient undergoing microsurgical breast reconstruction, important intraoperative anatomical considerations related to positioning and padding (Figure 2), as well as recommendations for pre-, intra-, and postoperative measures to reduce the risk of PI in these patients.

## Risk factors for intraoperative PI development in patients undergoing microsurgical breast reconstruction

### External risk factors

The most important risk factors for intraoperative PI development are the patient's immobility and inability to perceive pain secondary to general anesthesia and paralysis. Continuous, unrelieved, sustained pressure load and deformational changes on the skin and soft tissues over bony prominences during an operation lead to tissue ischemia and resulting PI (2). Intraoperative hypotension and hypoxia related to induction of general anesthesia result in hypoperfusion and tissue hypoxemia (5, 13). Hypovolemia and hemorrhage resulting in hypotension and/or need for vasopressors are associated with increased risk of PI (5).

### Length of surgery

The length of surgery is the most important risk factor (14), but perhaps even more important to note is that the total immobility time is always at least 3–4 h longer than the actual surgical time (including pre- and postoperative immobility time) (5, 13, 15). The risk of PI following a 3 h surgery is approximately 6%. When surgical time extends beyond 4 h, the risk of PI increases to 9% and for operations lasting >7 h, the incidence exceeds 13% (2, 5, 13). Moreover, when surgery lasts greater than 4 h, the risk of PI increases by 33% with every additional 30 min of surgical time (4). Even in the most experienced hands and most efficient surgical systems the total surgical time for microsurgical breast reconstructions exceeds 3 h in the vast majority of cases (8, 11, 12). Moreover, changes in patient positioning are required in nearly all cases, increasing the risk of patients sliding out of proper positioning and padding. Therefore, we find it helpful to mention these risk factors during the surgical time out. This ensures that all staff members are aware of the patient's risk of PI and allows an opportunity to remind staff members to verify positioning intermittently throughout the case at pre determined time points. We find it helpful to also include in the surgical time out the plans for anesthesia and nursing staff to verify positioning every 2–3 h during the procedure, as well as immediately after any changes in operating table position. It should also be included in pass-off between nursing and anesthesia staff when the last and when the next positioning checks should be performed.

### Temperature

Patients under general anesthesia and undergoing surgery are at increased risk of hypothermia due to a combination heat loss to the environment as well as inhibited behavioral and thermoregulatory responses (2, 5). Operating rooms are generally kept from 68 to 75°F (20–24.4 °C) and patient warming devices employing convection air or warmed fluids are typically used to keep the patient's goal body temperature >36 °C. A drop in core body temperature of 1.8 °C below normal results in elevated metabolic demands to restore normal body temperature and an associated 20% increased risk of PI (16). Elevated body temperature also results in a 10% increase in metabolism for each 0.6 °C rise in temperature (16). Above 38.1 °C the risk of perspiration lends to a significantly increased the risk of PI as it may result in friction and shearing forces on the skin (5). Typically, patient warming devices exist in either immersion type (water circulating) or forced air. They may be placed under or over patients. Immersion devices are more likely to result in perspiration and as a result moisture. Devices that are placed under the patient may be more likely to result in pressure points or shearing forces, especially with any changes in position (5). Forced air circulating warming devices placed over the patient are generally preferred in the operating room; however, in the context of abdominally based free flap reconstructions, they can only be used over the lower extremities and in the case of thigh based free flap reconstructions, they may not be used at all. Maintaining thermoregulation is a priority, therefore underbody warmers are typically preferred in microvascular cases where large surface areas are exposed for simultaneous access of distant donor and recipient sites. Emphasis should be placed on maintaining normal core body temperature and avoiding perspiration of the patient throughout the case. When warmers placed beneath the patient are used, we suggest verbalizing this as a risk for sliding and shearing in the surgical time out to emphasize the importance of verifying correct positioning especially after changes in the position of the operating room table.

### Positioning, positioning devices and prophylactic dressings

#### Positioning

In immediate microsurgical breast reconstruction, there is frequently a breast surgery team and reconstructive surgery team working simultaneously. The operating room table is typically positioned with the anesthesia team at the head of the bed; however, surgeons may opt to turn the operating room table 180° to better facilitate concomitant surgical teams (i.e., breast cancer surgeons working on mastectomy, axillary node sampling and reconstructive surgeons working on flap harvest). In circumstances where the bed is turned, anesthesia staff and operating room nurses should be particularly attentive to pressure points at the head, neck, and face as they are not as closely monitored in this position. Clear sterile drapes to maintain complete visualization of the head and neck are helpful especially in this position.

In abdominally based microsurgical breast reconstruction, patients are typically placed supine on the operating room table and later placed in a semi-recumbent position, flexed at the hip. For thigh based free flap reconstructions the patient may be placed supine in frog-legged position or prone, whereas for gluteal based flap harvest the patient will be in the lateral decubitus or prone position (17). In the supine position, the patient is at risk of PI to the occiput, scapulae, hips, sacrum/coccyx, and heels (13). Prone positioning results in increased risk of PI on the forehead, chin, chest/breasts, anterior shoulders, iliac crests, knees, shins, and toes (13). In the lateral position the bony prominences around the hip, shoulder, axilla, and ankles are most at risk of PI if not well positioned or padded (18). Additional concerns in the prone position is the risk of direct compression of the globes resulting in increased intra-ocular pressure, impaired retinal perfusion, and subsequent permanent vision loss. Compression neuropathies of the lateral femoral cutaneous nerve, ulnar nerve, and injury to the brachial plexus are also at greater risk in patients in the prone position compared to the supine position (13).

With the patient in the supine position, the patient may have their arms extended to 90° on arm boards or tucked. If the arms are tucked, all identification/allergy bands must be removed from the upper extremities and all intravenous tubing must be appropriately padded and secured prior to tucking the patient's arms. Kerlix, Webril rolls, or foam padding may be placed within the hands to keep the fingers and wrist in neutral position. Pressure points along the elbow and wrists should be appropriately padded, paying careful attention to the medial epicondyle of the elbow for risk of ulnar nerve compression (Figure 2).

Patients undergoing abdominally based free flap reconstruction are generally sat up on the operating room table to facilitate abdominal closure and flap inset in a routine manner. During this time, the hips are flexed, and the back is raised. It is important that the patient's hips are flexed (i.e., legs are raised) first, and then the head of the bed is elevated, to prevent shearing injury from the sliding. Any changes in OR table positioning increase the patient's risk of PI, as the patient may shift on the table. Any pressure areas that were previously well padded may become at greater risk of PI after shifting and prone to shearing and friction from the position changes. For unilateral gluteal-based flap harvesting, the patient may be placed in lateral decubitus position for the entire procedure, avoiding the risks of shearing and sliding with position changes (18). For bilateral procedures, the patient is prone for flap harvesting and then supine for the microsurgical anastomosis and flap inset (19). For thigh-based flap harvest the patient may be in frog-leg position with the hip and knee flexed or in lithotomy. Donor site closure may be difficult even in the frog-leg position, and leg elevation by an assistant is sometimes helpful for the posterior closure (11, 17, 20).

**Figure 3:**
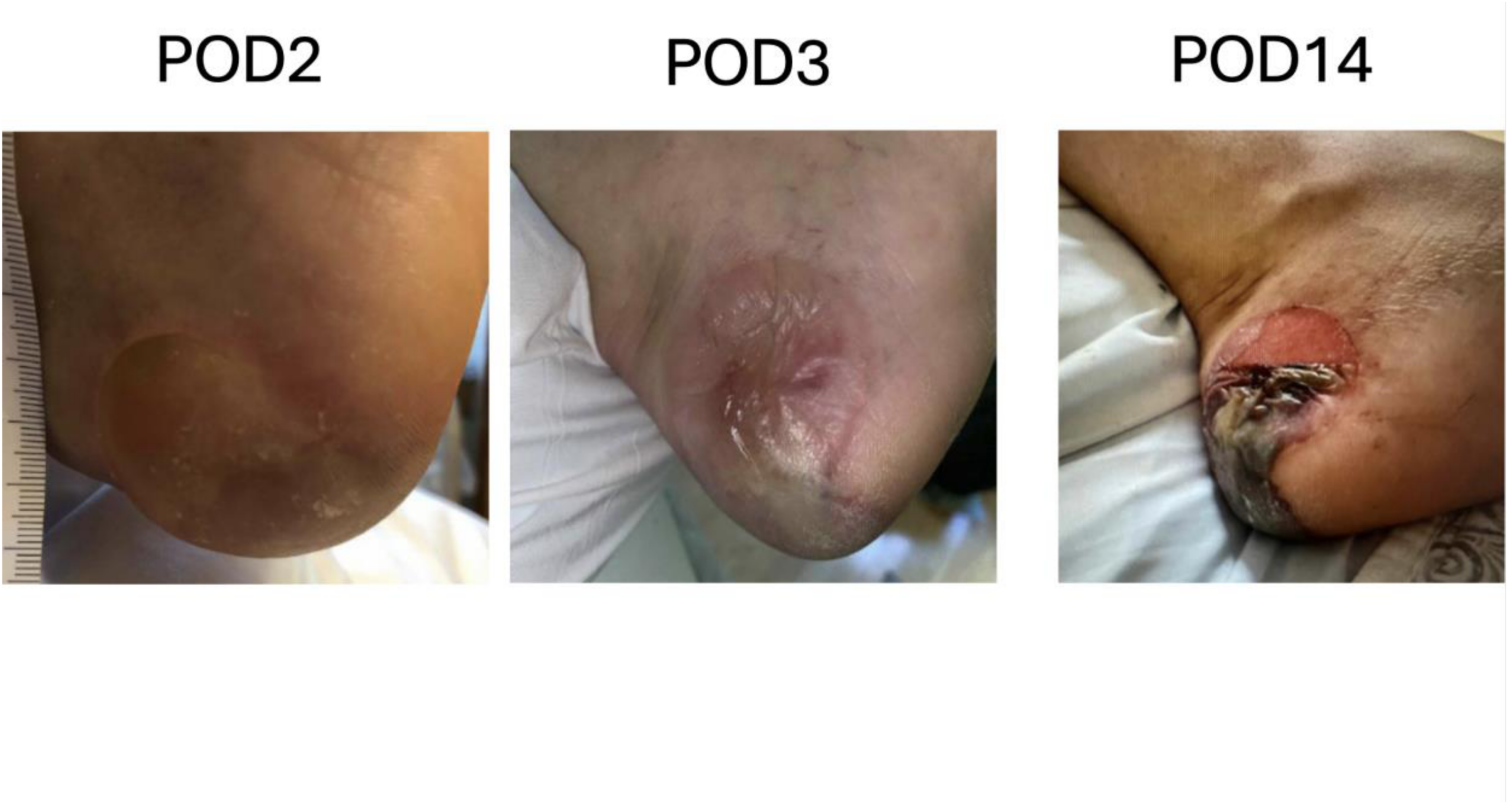
Example of a case of pressure injury to the left heel after a bilateral deep inferior epigastric artery perforator flap.

As these changes in positioning are expected, surgical teams should communicate, anticipate, and verify positioning of high-risk pressure points. The patient's positioning should be verified after any change in position of the operating room table. Additionally, the circulating nurse should verify positioning under the drapes and perform small repositioning s as necessary every 2–3 h, to ensure there is no inadvertent shifting of the patient into positions that are high risk for PI. This is especially important for the heels (Figure 3), which should be floating, and for the hands which are at risk of compartment syndrome if an IV infiltration were to occur and be undiagnosed.

#### Positioning devices

Operating room tables should be equipped with polyurethane or polyether mattresses that significantly reduce pressure compared to standard OR table mattresses (2, 21). Additionally, gel pad or foam pad overlays have been reported to reduce the probability of PI (5, 16). Linen on the operating room table should be free of any wrinkles and there should be no fluid pooling under the patient. Many different types of positioning devices exist, composed of various materials. Foam and gel devices offer limited protection against pressure injury, as they are considerably stiffer than supported soft tissues. They are relatively non-conforming, and therefore offer limited immersion and envelopment of bony prominences when compared to fluidized positioners. These more traditional gel or form products transfer pressure onto adjacent soft tissues, but do not necessarily disperse or absorb pressures. In the case of gel donuts, the pressure is redistributed to the ring around the donut on the scalp. Regardless of the type of donut (gel or foam) used, we recommend rotating the head from side to side every 2–3 h to reduce the risk of PI and pressure-induced alopecia (22). While some advocate for periodic scalp massage in the prevention of occipital pressure injury and pressure-induced alopecia, the evidence supporting this practice is lacking (22–24). Moreover, the repeated use and sterilization of any gel or foam products reduces their resilience and recoil (5). The more novel fluidised positioners have been designed to maximally envelope soft tissues around bony prominences most at risk of PI. They may be molded into any shape and then under the sustained pressure of body weight will maintain that shape and redistribute the weight onto the largest possible contact area along the fluidized positioning device (5). They therefore are better able to efficiently redistribute pressures, have lower peak and lower average interface pressure, making them superior devices for prevention of PI (25).

Special attention should also be given to the placement of the endotracheal tube, orogastric tube, temperature probes, and bis monitors placed along the head and neck. Communication with anesthesia team should be clear to avoid the risk of PIs along the nares, lips, forehead, ears or anywhere else they may have monitors placed. Endotracheal tube and ventilator tubing should be directed away from the surgical field. To accommodate the tubing toward the direction of the surgical field and avoid PI on the face, create a foam halo using a foam donut, in which the ventilator tubing may be secured safely without risk of PI. We also use a clear sterile drape at the head of the bed, for the patient's face and neck to be fully visualized by the surgeons at all times during the operation.

#### Prophylactic dressings

The sacrum and heels are at the highest risk of PI, both when the patient is in supine and recumbent positions. Multilayered silicone foam dressings have been demonstrated to reduce shearing and friction forces and significantly reduce the incidence of pressure injuries when applied to the sacrum and heels (26, 27) and are therefore recommended prophylactically to these areas in patients at high risk of developing PIs (28, 29).

### Intrinsic risk factors

Intrinsic risk factors to PIs include nutritional status (albumin <3 g/dl, BMI <18), age (>60), baseline impairment in mental status or mobility, incontinence, infection, existing PI and comorbidities (diabetes mellitus, peripheral arterial disease, chronic obstructive pulmonary disease, obesity, chronic kidney disease, anemia) (2). Patients with diabetes mellitus have two-fold risk of developing intraoperative PI (16). Patients undergoing microsurgical breast reconstruction generally would have few of the risk factors, and any existing intrinsic risk factors, such as diabetes mellitus or anemia, would be optimized prior to surgery. Consultation with patient's primary medical doctor, endocrinologist, and/or hematologist is recommended to ensure that hemoglobin A1c and hematocrit are optimized with as needed prescription medications (including forms of insulin), iron supplementations, red blood cell transfusions etc. (Table 1).

**Table 1:** Pressure injury (PI) risk factors and risk assessment tools (4).

Preoperative, intraoperative and postoperative PI risk factors	
Intrinsic	Extrinsic
Preoperative and postoperative	Preoperative
Existing pressure Braden score	Previous surgery
Older age (>60)	Intraoperative
Malnutrition (albumin <3 g/dl, BMI <18)	Length of surgery (>3 h) Odds of PI = 1.6× for 4–6 h Odds of PI = 6.4× for >6 h *Each +*1 h > 6 h *increases risk of PI* > 10%
ASA (>2) Comorbidities (Diabetes mellitus, peripheral arterial disease, chronic obstructive pulmonary disease, obesity, hypotension/vasopressor requirements, CKD, anemia)	Position (prone, supine, lateral, etc.)
Decreased mental status, impaired sensation	Positioning (floating heels)
Impaired mobility vs. immobility	Positioning devices used (gel pressure overlays)
Infection	Hypothermia, warming devices (forced-air vs. immersion)
Incontinence	estimated blood loss, hypotension and vasopressors
	Moisture/shearing (perspiration, position changes/sliding)

PI risk assessment tools			
Risk assessment tool	Contributing patient clinical factors	Advantage	Disadvantage
Braden scale	Considers patient's ability to perceive sensation and their mobility, exposure to moisture (such as incontinence), friction and shear, and nutritional status.	Typically performed preoperatively.	Does not require a skin exam be performed.
Munro scale	Considers mobility, nutritional status, BMI, recent weight loss, age and comorbidities at the preoperative stage. Intraoperatively factors including physical status, ASA score, anesthesia type, body temperature, hypotension, moisture, surface/motion and position are considered. Post-operatively the Munro scale considers the length of procedure and total blood loss.	The total Munro scale calculates the cumulative score and classifies patients as low, medium and high risk. Used at each perioperative phase (pre-, intra- and post-operatively). Requires nurses to document their hand offs as part of the assessment.	Lengthy, but thorough.
Scott-Triggers	Considers a patient high risk when 2 or more factors are present pre-operatively: age > 62 or older; serum albumin of <3.5 g/L or a BMI < 19 or >40, ASA score of 3 or more, and OR time >180 min.	Associated with annual $1,000.00 copyright fee.	Performed preoperatively and triggers a set of evidence-based practices that should be implemented based on risk (i.e offloading of heels)

## Preoperative risk and postoperative protocol

Several validated measurements exist for predicting the risk of perioperative PI, including the Braden Scale, the Munro Scale, and the Scott-Triggers assessment (Table 1) (4, 16). There is little evidence to support one tool over the others and the predictive power of each one is limited if not used with appropriate clinical judgment. However, they are helpful in assessing the patient's risk for developing PI accounting for intrinsic and extrinsic risk factors at specific perioperative times. Quality improvement studies have demonstrated that when nursing documentation has included PI risk assessment in the electronic medical record, nursing satisfaction and communication is improved, and the incidence of perioperative PI remains low (30). A well-documented pre-operative and postoperative skin exam, as well as well clear documentation of the patient's preoperative risk factors and risk level are essential for an informed handoff between nurses. Postoperatively early ambulation is key to reducing risk of perioperative PI. At our institutions all patients are out of bed on post-operative day 0 with assistance and ambulating on postoperative day 1 (Table 2).

**Table 2:** Practices to reduce the risk of PI in patients undergoing microsurgical breast reconstruction.

General preoperative skin and risk assessment	1. Standard skin assessments based on institutionally preferred risk assessment scale 2. Clear documentation of patient risk factors, risk level and skin exam 3. Standard hand-off among preoperative and operative nurse 4. Multilayer foam dressings placed on sacrum and heels (i.e., Mepilex® Border Sacrum and Heel dressings)
Intraoperative procedures	1. Pad all pressure points with fluidized positioners (preferred) 2. If fluidized positioners are not available, judicious use of gel/foam positioning devices for offloading of pressure points, namely occiput, elbows, wrists, and heels 3. Review and verify positioning paying attention to key anatomical areas (as described in Table 2 and Figure 1) 4. During the surgical time out, review patient's risk for PI and plan to rotate head, verify positioning (with special attention to wrists and heels) every 2–3 h and after every change in position.
Postoperative enhanced recovery pathways	1. Encourage mobility out of bed, even if to the edge of the bed with assistance only, on postoperative day 0 2. Enhanced recovery after surgery pathways that encourage patients early ambulation should be implemented to reduce continued unrelieved pressure in the supine/seated position

## Conclusion

Intraoperatively acquired PI are a significant cause of preventable patient morbidity and health care costs. Surgical time is one of the greatest risk factors for PI development, with patients undergoing surgeries with operative times >3 h at the most elevated risk. Additionally, changes in patient positioning on the OR table also increase the risk or PI, making surgical PI an important concern for reconstructive plastic surgeons and patients undergoing microsurgical breast reconstructions. Herein, we have proposed a set of simple practices aimed at reducing the risk of PI in patients undergoing microsurgical breast reconstructions with that they may benefit surgeons and patients beyond our institution.
